# Understanding health policy leaders’ training needs

**DOI:** 10.1371/journal.pone.0174054

**Published:** 2017-03-23

**Authors:** Carey Roth Bayer, L. Lerissa Smith, Renée Volny Darko, Marissa McKool, Fengxia Yan, Harry Heiman

**Affiliations:** 1 Satcher Health Leadership Institute, Morehouse School of Medicine, Atlanta, Georgia, United States of America; 2 Departments of Community Health and Preventive Medicine/Medical Education, Morehouse School of Medicine, Atlanta, Georgia, United States of America; 3 Department of Community Health and Preventive Medicine, Morehouse School of Medicine, Atlanta, Georgia, United States of America; 4 Department of Family Medicine, Morehouse School of Medicine, Atlanta, Georgia, United States of America; TNO, NETHERLANDS

## Abstract

**Purpose:**

We assessed the training needs of health policy leaders and practitioners across career stages; identified areas of core content for health policy training programs; and, identified training modalities for health policy leaders.

**Methods:**

We convened a focus group of health policy leaders at varying career stages to inform the development of the Health Policy Leaders’ Training Needs Assessment tool. We piloted and distributed the tool electronically. We used descriptive statistics and thematic coding for analysis.

**Results:**

Seventy participants varying in age and stage of career completed the tool. “Cost implications of health policies” ranked highest for personal knowledge development and “intersection of policy and politics” ranked highest for health policy leaders in general. “Effective communication skills” ranked as the highest skill element and “integrity” as the highest attribute element. Format for training varied based on age and career stage.

**Conclusions:**

This study highlighted the training needs of health policy leaders personally as well as their perceptions of the needs for training health policy leaders in general. The findings are applicable for current health policy leadership training programs as well as those in development.

## Background

Health and health care leaders are increasingly recognizing the importance of health policy and health policy training. This growing consensus has also been reflected in a number of reports over the past 10-15 years. The Institute of Medicine in its 2003 report, *Who Will Keep the Public Healthy*? *Educating Health Professionals for the 21st* Century[1], stressed the importance of both politics and policy to the future of public health and health care. In their 2010 report, the Commission on Education of Health Professionals for the 21st Century[2] noted the importance of planning, policy, and management for future health leaders. As the interest and importance continues to grow, so does the opportunity for adding health policy into existing training structures.

Medical, nursing, and public health leaders and educators have advocated for the importance of health policy training to support engagement and leadership in public policy issues impacting their profession and the health of communities they serve.[3–8] While health policy training is increasingly being incorporated into health professional training programs, a number of challenges and barriers have been identified. These include scheduling and time constraints[9], lack of perceived relevance, lack of or competition for limited resources, and lack of faculty expertise and interest.[10] One of the most formidable barriers for programs implementing health policy training is the lack of meaningful data and research to inform the teaching and implementation of health policy training.[11] As a result, there is little consensus or evidence to inform best practices with regard to the desired training goals and learner outcomes, core content, or teaching methods. This lack of clear training goals and measureable outcomes makes meaningful evaluation, beyond self-reporting of learner outcomes and learner satisfaction, virtually impossible.

At a time when health professionals are increasingly aware of the value and importance of health policy, there is a compelling need for more research to inform health policy training. The purpose of this study was to assess the training needs of health policy leaders and practitioners at different career stages; to identify areas of core content for health policy training; and, to identify the most effective training modalities for health policy leaders.

## Methods

We received Institutional Review Board (IRB) approval from the Morehouse School of Medicine (MSM) IRB to conduct this work. In February and March, 2013, we recruited health policy leaders and practitioners across career stages to assist in the development of the health policy leaders’ training needs assessment tool. We recruited through electronic invitation and word of mouth and indicated that the purpose of the group was to identify the content to include in a health policy leadership training needs assessment tool. We used purposive sampling to insure the group would have relevant background and experience with health policy. The multidisciplinary group consisted of graduates from two of MSM Satcher Health Leadership Institute’s (SHLI) training programs, the Health Policy Leadership Fellowship Program and Community Health Leadership Program, and professionals working in public health, health advocacy, and health policy. The group included nine individuals (five women, four men, one transgender man) from four states and the District of Columbia with a racial/ethnic breakdown of six Black/African-American and three White/Caucasian individuals. Educationally, they ranged from college graduate to doctoral graduate as well as from recent graduate/early career professional to late career professional.

In March, 2013, we convened the group for an all-day, face-to-face focus group/tool development session. We divided the group by career level with respect to health policy leadership experience - student, early career professional, mid-career professional, late career professional; and, asked open ended questions. We intentionally divided by career level for the discussion to make sure the final needs assessment tool would be applicable across the career spectrum. To help frame the discussion, we asked participants to describe or define the term “health policy leadership” before moving into discussions about health policy leaders’ training needs. In the context of considering the training needs of health policy leaders, we asked them to answer the following questions:

1. What knowledge do health policy leaders need to be effective?
2. What skills do health policy leaders need to be effective?
3. What attributes do health policy leaders need to be effective?
4. What are the best formats in which to train health policy leaders?
5. What should be considered in developing and disseminating a needs assessment tool for health policy leadership training?

Each small group reported out during the large group discussion. New graduates discussed “student” topics due to their proximity to the formal education system. Where there were differences of perspective, the group came to a consensus on the point. We compiled the results of the focus group and qualitatively analyzed the responses to establish the needs assessment tool sections and elements. Commonalities existed across the small groups and we reached saturation for developing the tool items. In April and May, 2013, we developed the draft needs assessment tool.

The needs assessment tool consisted of general demographic questions and both open and closed-ended questions covering the focus group-identified areas of health policy leaders’ knowledge, skills, and attributes as well as training formats. After vetting and piloting the draft tool with the focus group of health policy professionals and members of the MSM Research and Evaluation Cores – professionals who specialize in research methodology and analysis as well as evaluation metrics —, we revised the tool based on the feedback received. We formatted the tool for electronic distribution using SurveyMonkey, an online survey platform. Closed-ended questions required respondents to consider their personal training needs as well as those of health policy leaders in general, using a 5-point Likert scale ranging from unimportant to critical. The attributes section asked participants to rank order the top 7 attributes, again from the perspectives of both their personal training needs and health policy leaders’ training needs in general.

We calculated the frequencies and percentages for each question and summarized them. We also described the top 5 selected Knowledge Elements, top 10 selected Skill Elements and the most frequently selected 7 Attribute Elements for importance to personal development as a health policy leader as well as importance to the development of health policy leaders in general.

We disseminated the tool from October to December, 2013, inviting individuals with health policy interest as well as those involved in health policy leadership to complete the tool. Given the limited information published on health policy training and the study team’s knowledge and health policy career networks, our recruitment efforts included multiple electronic prompts to participate at repeated intervals with 26 universities, six Facebook Groups, nine LinkedIn Groups, 12 professional organizations, health policy fellowship training program alumni, including the Robert Wood Johnson Health Policy Fellows Program and SHLI Health Policy Leadership Fellowship Program, and through word of mouth. All of the institutions, social media groups, and fellowship programs had health policy connections. We aimed to recruit in places likely to have traffic from individuals interested in health policy or engaged in health policy careers. In this IRB approved study, participants gave electronic informed consent to participate in the survey on the first page of the survey in SurveyMonkey. Since the survey was anonymous, electronic participation served as documentation of consent.

## Results

We had 70 diverse individuals complete the health policy leaders’ training needs assessment tool. (Table 1) Participants ranged in age from 25 years to greater than 65 years and included slightly more females than males or transgender individuals. The participants were predominantly either Caucasian/White or African American/Black. The demographic outcomes that were most surprising included the education levels (75%, n = 46 with earned doctoral degrees) and income levels (50%, n = 30 with a household income over $100,000/year) of the participants.

**Table 1:** Demographics Characteristics of Participants.

Characteristic	Total, % (n)
**Gender**	
Female	54 (33)
Male	44 (27)
Transgender	2 (1)
**Age** (in years)	
25-35	18 (11)
36-45	12 (7)
46-55	18 (11)
56-65	19 (12)
>65	33 (20)
**Highest education level**	
Bachelor's Degree	7 (4)
Master's degree	18 (11)
Doctoral degree	75 (46)
**Race**	
African American/Black	21 (13)
Asian/Pacific Islander	2 (1)
Other	3 (2)
Caucasian/ White	74 (45)
**Hispanic or Latino origin**	
Yes	2 (1)
No	98 (59)
**Household income**	
$10,000-$25,000	5 (3)
$25,001-$40,000	0 (0)
$40,001-$55,000	2 (1)
$55,001-$75,000	10 (6)
75,001-$100,000	12 (7)
Over $100,000	50 (30)
Prefer not to disclose	22 (13)

* Due to missing data not all categories have an N = 70

For the purposes of this analysis, we collapsed the 5-point Likert scale into a 3-point scale (unimportant/slightly important, important/very important, critical) for the knowledge items and the skill items. Top ranked areas of knowledge, skills, and attributes are shown in Tables 2–4 as described below. Rank ordering reflects only the critical category; we included corresponding percentages for important/very important and unimportant/slightly important for purposes of comparison.

**Table 2:** Top 5 Selected Knowledge Elements.

**Importance to my personal development as a health policy leader**			
**Variable**	**Unimportant/slightly important % (n)**	**Important/very important % (n)**	**Critical % (n)**
Cost implications of health policies	4 (3)	33 (22)	63 (42)
Intersection of policy and politics	6 (4)	37 (25)	57 (38)
Local, state, and federal legislative processes	1 (1)	51 (34)	48 (32)
Policy analysis	2 (1)	66 (43)	32 (21)
Role of research in policy development	7 (5)	66 (44)	27 (18)
**Importance to the development of health policy leaders as a whole**			
**Variable**	**Unimportant/slightly important % (n)**	**Important/very important % (n)**	**Critical % (n)**
Intersection of policy and politics	1 (1)	45 (30)	54 (36)
Local, state, and federal legislative processes	2 (1)	58 (39)	40 (27)
Policy analysis	0 (0)	66 (42)	34 (22)
Breadth and scope of health policy	0 (0)	71 (47)	29 (19)
Dimensions of good policies	0 (0)	72 (47)	28 (18)

*Due to missing data not all categories have an N = 70

Top 5 ranking was calculated only for knowledge categories identified as critical

Some percentages are not consistent with the frequencies due to missing values

**Table 3:** Top 10 Selected Skill Elements.

**Importance to my personal development as a health policy leader**			
**Variable**	**Unimportant/slightly important % (n)**	**Important/very important % (n)**	**Critical % (n)**
Effective communication skills	0	37 (23)	63 (39)
Critical thinking skills	0	47 (29)	53 (33)
Engaging decision makers	2 (1)	45 (28)	53 (33)
Engaging stakeholders	2 (1)	48 (30)	50 (31)
Leadership skills	2 (1)	59 (36)	39 (24)
Garnering political support	10 (6)	53 (33)	37 (23)
Cultural competency skills	3 (2)	61 (38)	35 (22)
Crafting health policy messages	7 (4)	61 (37)	33 (20)
Presentation skills	2 (1)	66 (40)	33 (20)
Advocacy skills	6 (4)	61 (38)	32 (20)
Negotiation skills	6 (4)	61 (38)	32 (20)
**Importance to the development of health policy leaders as a whole**			
**Variable**	**Unimportant/slightly important % (n)**	**Important/very important % (n)**	**Critical % (n)**
Effective communication skills	0	37 (23)	63 (39)
Engaging stakeholders	0	43 (27)	57 (36)
Engaging decision makers	0	46 (29)	54 (34)
Critical thinking skills	0	49 (31)	51 (32)
Garnering political support	2 (1)	56 (35)	42 (26)
Leadership skills	0	60 (36)	40 (24)
Cultural competency skills	3 (2)	59 (37)	38 (24)
Assessing strengths and weakness of various policies	0	65 (41)	35 (22)
Crafting health policy messages	2 (1)	63 (39)	35 (22)
Negotiation skills	2 (1)	66 (41)	32 (20)
Networking skills	3 (2)	65 (40)	32 (20)

* Due to missing data not all categories have an N = 70

Some percentages are not consistent with the frequencies due to missing values

**Table 4:** The Most Frequently Selected 7 Attribute Elements.

	Important to my individual training needs as a health policy leader (%)	Important to training needs of health policy leaders as a whole (%)
Rank	Attribute (%)	Attribute (%)
1	Integrity (41)	Integrity (43)
2	Professionalism (32)	Professionalism (35)
3	Perseverance (30)	Being organized (34)
4	Passion (27)	Open mindedness (32)
5	Open mindedness (26)	Motivation (28)
6	Being organized (25)	Perseverance (25)
7	Motivation (23) or Empathy(23)	Passion(22) or Empathy (22)

Raw sample size not included as participants ranked top 7 attributes from a list of 24

Table 2 lists the top five knowledge elements selected as critical for the development of health policy leaders. The five most frequently selected for personal development were: cost implications of health policies (63%, n = 42); intersection of policy and politics (57%, n = 38); local, state, and federal legislative processes (48%, n = 32); policy analysis (32%, n = 21); and role of research in policy development (27%, n = 18). By comparison, the five most frequently selected knowledge elements for health policy leaders in general were: intersection of policy and politics (54%, n = 36); local, state, and federal legislative processes (40%, n = 27); policy analysis (34%, n = 22); breadth and scope of health policy (29%, n = 19); and dimensions of good policies (28%, n = 18).

Skill building results, as shown in Table 3, include the top 10 skills identified as critical for both personal development and development of health policy leaders in general. For personal development, participants deemed effective communication skills the most critical at 63% (n = 39), followed by critical thinking skills and engaging decision makers, both of which tied at 53% (n = 33); engaging stakeholders followed with 50% (n = 31); leadership skills with 39% (n = 24); garnering political support with 37% (n = 23); cultural competency skills with 35% (n = 22); crafting health policy messages and presentation skills tied at 33% (n = 22); and, both advocacy and negotiation skills at 32% (n = 20).

For importance to the development of health policy leaders as a whole, participants deemed effective communication skills the most critical at 63% (n = 39) followed by engaging stakeholders at 57% (n = 36); engaging decision makers at 54% (n = 34); critical thinking skills at 51% (n = 32); garnering political support at 42% (n = 26); leadership skills at 40% (n = 24); cultural competency skills at 38% (n = 24); assessing strengths and weaknesses of various policies and crafting health policy messages, both at 35% (n = 22) and, both negotiation and networking skills at 32% (n = 20).

We listed the most frequently selected attributes for both personal and general training needs in Table 4. In rank order, the top 7 attributes selected as important for personal development included integrity (41%), professionalism (32%), perseverance (30%), passion (27%), open mindedness (26%), and being organized (25%), with both motivation and empathy at 23%. As for importance to general training needs, integrity and professionalism remained the top two selected attributes (at 43% and 35%) followed by professionalism (35%), being organized (34%), open mindedness (32%), motivation (28%), and perseverance (25%), with passion and empathy at 22%.

When asked about format for engaging in health policy training, participants selected conferences (27%, n = 16) as “would definitely participate”, and, retreats (32%, n = 18) as “likely to participate.” The two formats participants most frequently selected as “would not participate” included traditional multi-year degree training (62%, n = 37) and paper-based self-directed learning (58%, n = 35). Seven participants gave qualitative responses explaining that at their stage of career, they were not be inclined to go through further intensive training. One participant said, “I am well into my career at this point so I am not likely to do more intensive training, but in the past I would have liked to have these opportunities [referring to the list of training options included in the tool].” Two other themes that arose from the qualitative comments related to determining the likelihood of participation included the costs associated with the training options as well as the content/speakers.

## Discussion

To the best of our knowledge, this is the first study specifically designed to begin assessing health policy leaders’ training needs. It is clear that many factors need to be considered, including training content and delivery, learner age and stage of career, financial capacity, self-perceptions of training needs, and career motivations. While there was clear overlap in perceived self needs compared to perceptions of the broader needs of health policy leaders in general, in this sample, there were also notable differences. “Effective communication skills” ranked highest for the participants’ personal skill development as well as for health policy leaders on the whole, implying recognition of the critical role of communication in health policy. Similarly, “integrity” and “professionalism” were ranked highest as both personal needs and needs in general. Participants ranked “cost implications of health policies” as most critical for personal knowledge development, yet this same item did not appear among the top 5 as a critical element for health policy leaders in general. It is not clear why this group of participants viewed cost implications as less of a priority for health policy leaders in general. Participants also ranked “presentation skills” and “advocacy skills” in the top 10 skills as personal needs; yet, neither of those made the top 10 for health policy leaders in general. Participants ranked “assessing strengths and weaknesses of various policies” and “networking skills” in the top 10 skills for health policy leaders in general; yet, neither of those made the top 10 for personal development. Given that our sample contained a large portion of doctorally-prepared participants, it is possible that formal training and stage of career impacted their rankings.

Several questions emerged during our analysis including: why were there discrepancies between self-perception and perception of general needs?; what role do age, stage of career, and lived experience play in developing formal health policy training program curricula?; and, how do we measure the effectiveness of health policy leadership training programs given the complexity of knowledge, skill, and attribute needs? The answers to these questions as well as the results from this study may spark new thinking about intentional training in health policy leadership across a variety of disciplines including medicine, nursing, public health, law, political science, psychology, and others. Given the limited evidence to inform the development of health policy training programs[11–12], future research addressing these questions may help to set benchmarks or desired outcomes for such programs.

## Limitations

We intentionally did not define “health policy leader” and “leadership,” which may have influenced potential participants’ decisions to participate in the study. Since we wanted to understand health policy leaders’ training needs and perceptions, we intentionally did not go into exhaustive operational definitions for most elements in the tool, which may also have influenced participants’ responses. In spite of widespread electronic marketing and recruitment efforts, the final sample remained small. Our sample included a large number of doctorally-prepared participants and a third of participants were 65 years or older, which may indicate that having the word “leader” in the title and tool did not resonate with those at earlier stages of their careers. It is not clear at what point one self-labels as a “leader” or “expert” and those who are not comfortable embracing those labels may have self-selected not to participate. Understanding at what career stage an individual self-identifies as a “leader” or “expert” in health policy may help explain our sample size. It is possible that many professionals who work on health policy do not self-identify as “leaders” despite engaging in leadership roles. Though we organized the tool to better understand knowledge, skill, attribute, and training format needs, as one participant noted, “I’m not sure how you ‘train’ integrity or selflessness or humility,” which raises the question of how feasible it is to measure attributes in health policy leadership training. The findings represent the perspectives of the study group and their specific health policy interest and career experiences.

## Conclusions

Based on the findings from this study, our next steps include deeper analyses to determine further distinctions in knowledge, skill, and attribute needs related to career stage. We also plan to map the top knowledge, skill, and attribute items to the SHLI Health Policy Leadership Fellowship Program curriculum to assess overlap and gaps in training health policy leaders. As disciplines across healthcare (e.g. medical, public health, nursing) have cited a need for health policy leadership[3–8,12], our assessment helps to begin building a foundation of evidence to inform the development of such trainings.

Very little exists in the literature on specific health policy training programs and learner outcomes and impact[12]. While this study provides valuable information regarding health policy training needs from a convenience sample of professionals engaged in health policy, it does not address a number of critical issues regarding health policy training looking forward – specifically: How does health policy training intentionally move beyond “healthcare” policy to adopt a health in all policies lens and approach? How can training programs intentionally engage diverse learners across disciplines, sectors, and demographic groups to enrich the learning experience and prepare the needed workforce? Last, how can health policy training programs integrate health equity across the fabric of training programs to ensure that developing health policy professionals and leaders are prepared to advance policies and practices that ensure opportunities for optimal health for all people? This study begins to shed light on the possible content needed in health policy training and reiterates the importance of intentional leadership training for ensuring future generations are prepared to leverage health policy to improve health outcomes and advance health equity.
